# Focus on blood cancers and liquid biopsy research

**DOI:** 10.1002/1878-0261.13073

**Published:** 2021-09-01

**Authors:** Ruzhica Bogeska

This issue of *Molecular Oncology* focuses on two key topics: blood cancers and liquid biopsy research. We pick the Blood Cancer Awareness month of September to feature the Review Article by Karen Keeshan and colleagues; providing a comprehensive picture of genetic, epigenetic, transcriptional and metabolic profiles of acute myeloid leukemia (AML) in paediatric and adult patients, while also discussing clonality and molecular signatures of stem cells and chemoresistant cells in AML [1]. Further Research Articles in this issue investigate specific molecular signatures of AML and other myeloid neoplasms. Indicatively, Andrés‐Zayas *et al*. used a 15‐gene panel to identify and characterize myeloid neoplasms with germline predisposition through next‐generation sequencing [2]. Moreover, Hellesøy *et al*. observed sex‐dependent differences in mutational and transcriptional profiles and *ex vivo* drug responses of a total of 1755 patients with AML, therefore recommending sex‐adjusted clinical management of this heterogeneous disease [3].

Liquid biopsy is a valuable tool for diagnosis, patient stratification and treatment follow‐up of blood cancers and solid cancers alike, as also reviewed in a recent thematic issue of *Molecular Oncology* [https://febs.onlinelibrary.wiley.com/toc/18780261/2021/15/6]. Ongoing research aspires to address challenges related to testing sensitivity of liquid biopsy samples.

Tyrosine kinase inhibitors (TKIs) are currently being used for the treatment of patients with non‐small cell lung cancer (NSCLC) that carry *EGFR* mutations. However, during the course of treatment, patients often develop resistance to TKI therapies. Thus, mutation profiling of circulating tumour DNA (ctDNA) has been recommended in international guidelines and has entered clinical practice, in order to improve treatment decisions for patients with NSCLC. Unfortunately, ctDNA‐based tests show limited sensitivity in detecting *EGFR* mutations. The research of Luigi Pasini *et al*. [4] proposes the use of RNA contained in blood‐derived extracellular vesicles (EVs) for quantitative *EGFR* profiling in patients with NSCLC subjected to TKI treatment. The study employed longitudinal *EGFR* mRNA profiling in a representative cohort of patients treated with first‐ or second‐generation TKIs. The authors established a workflow for the ultrasensitive detection of *EGFR* mutations in circulating EVs and compared the data to previously established ctDNA‐based methods. Both methods adequately identified the type of *EGFR* mutations, where EV‐RNA profiling showed superior sensitivity in mutation detection. Additionally, changes in tumour EV burden were observed during disease progression and were associated with clinical features and disease outcomes in patients receiving TKI therapy. The study also suggests that EV‐RNA and ctDNA represent independent biological sources in liquid biopsy and that the molecular analysis of both could offer a more comprehensive understanding of tumour dynamics during NSCLC progression.

While the role of CTCs in cancer progression, as well as in clinical cancer management, is well established, reaching reproducible and adequate sensitivity of CTC detection and molecular profiling may represent a challenge for cancer diagnostics and treatment selection. Peng‐Xiang Wang and colleagues developed a new workflow for CTC enrichment, detection and single‐cell analysis [5]. The workflow was tested and validated in healthy donors and patients with different types of cancer. In summary, automated CTC enrichment at the ChimeraX‐i120 platform was combined with machine learning‐based CTC identification and single CTC sequencing. Apart from establishing the novel automated workflow for single CTC enumeration and molecular characterization in different cancer types, the authors employed the workflow in a cohort of patients with hepatocellular carcinoma (HCC) to evaluate the prognostic and clinical feasibility of their method. While the workflow yielded moderate diagnostic potential in distinguishing patients with HCC, it could be efficiently used for an accurate estimation of CTC numbers and as a complementary tool in screening and early diagnosis of patients with different types of cancer. Moreover, single CTC profiling could offer a comprehensive analysis of the tumour clonal composition and tumour heterogeneity before and after treatment.

In their article, Bade *at al*. [6] explore a novel strategy aimed at increasing the detection rate of circulating tumour cells (CTCs) in patients with advanced clear cell renal cell carcinoma (ccRCC). Peripheral blood CTCs were isolated and enumerated from a heterogeneous cohort of patients having various disease stages, metastasis sites and types of treatment. Captured CTCs were further characterized for several tumour‐specific markers. Analysis of the isolated CTCs demonstrated pronounced heterogeneity of CTC biomarker expression among patients, as well as a significant correlation of CTC numbers with clinical progression. Furthermore, longitudinal CTC characterization showed that CTCs could hold a pharmacodynamic potential for the evaluation of patients during therapy. The novel approach of CTC capture maximized the specificity of CTC identification and advanced the noninvasive evaluation of intra‐ and intertumoural heterogeneity. The authors propose deployment of these novel assays in prospective clinical trials to explore the clinical potential of their approach in patients with ccRCC.

## Conflict of interest

The authors declare no conflict of interest.
